# Multiple phosphorylation events control mitotic degradation of the muscle transcription factor Myf5

**DOI:** 10.1186/1471-2091-6-27

**Published:** 2005-12-01

**Authors:** Christine Doucet, Gustavo J Gutierrez, Catherine Lindon, Thierry Lorca, Gwendaline Lledo, Christian Pinset, Olivier Coux

**Affiliations:** 1 Centre de Recherches de Biochimie Macromoléculaire (CRBM), CNRS FRE 2593, Montpellier, France; 2 Present address: Burnham Institute for Medical Research, La Jolla, CA, USA; 3 Wellcome Trust/Cancer Research UK, Gurdon Institute, Cambridge, UK; 4 Celogos/Institut Pasteur, Paris, France

## Abstract

**Background:**

The two myogenic regulatory factors Myf5 and MyoD are basic helix-loop-helix muscle transcription factors undergoing differential cell cycle dependent proteolysis in proliferating myoblasts. This regulated degradation results in the striking expression of these two factors at distinct phases of the cell cycle, and suggests that their precise and alternated disappearance is an important feature of myoblasts, maybe connected to the maintenance of the proliferative status and/or commitment to the myogenic lineage of these cells. One way to understand the biological function(s) of the cyclic expression of these proteins is to specifically alter their degradation, and to analyze the effects of their stabilization on cells. To this aim, we undertook the biochemical analysis of the mechanisms governing Myf5 mitotic degradation, using heterologous systems.

**Results:**

We show here that mitotic degradation of Myf5 is conserved in non-myogenic cells, and is thus strictly under the control of the cell cycle apparatus. Using *Xenopus *egg extracts as an *in vitro *system to dissect the main steps of Myf5 mitotic proteolysis, we show that (1) Myf5 stability is regulated by a complex interplay of phosphorylation/dephosphorylation, probably involving various kinases and phosphatases, (2) Myf5 is ubiquitylated in mitotic extracts, and this is a prerequisite to its degradation by the proteasome and (3) at least in the *Xenopus *system, the E3 responsible for its mitotic degradation is not the APC/C (the major E3 during mitosis).

**Conclusion:**

Altogether, our data strongly suggest that the mitotic degradation of Myf5 by the ubiquitin-proteasome system is precisely controlled by multiple phosphorylation of the protein, and that the APC/C is not involved in this process.

## Background

Terminal differentiation of skeletal muscle is orchestrated by the family of myogenic regulatory factors (MRFs), which contains Myf5, MyoD, myogenin and MRF4 (for review see [1,2]). These factors activate muscle-specific gene transcription, by binding specific DNA sequences (E-boxes) as heterodimers with ubiquitous E2A proteins such as E12 and E47, in cooperation with MEF2 family of MADS-box proteins (reviewed in [3]). They have first been characterized for their ability to convert certain non muscle cells into myoblasts after ectopic expression, a process known as "myogenic conversion" [4,5]. Among MRFs, MyoD and Myf5 are usually considered as "determination factors" since they are required for formation of skeletal muscle [6], and expressed at the proliferating myoblast stage, in contrast to myogenin that is induced as cells undergo cell cycle arrest, and MRF4 that is involved in myotube maturation [7]. However, recent data have shown that MRF4 is also expressed at early stages of muscle development, and can act upstream of MyoD and Myf5 [8].

Interestingly, MyoD bears intimate functional relationships with the cell cycle apparatus (reviewed in [9]): its transcriptional activity and stability are regulated by cyclin/CDK complexes [10-13], and it can repress cell cycle activators by physical interaction [14] or by activating expression of cell cycle inhibitors [15]. Regarding Myf5, despite numerous observations about the regulation of its gene and expression pattern during embryonic development [7,16], little is understood about its functions, probably due in large part to its redundancy with MyoD [6].

However, very intriguingly, in cultured myoblasts (C2 cells or primary myoblasts), intracellular protein levels of Myf5 and MyoD exhibit opposite cell-cycle fluctuations at the proliferative stage [17], with the result that, during the early G1 phase of the cell cycle, MyoD levels are high and Myf5 levels low, whereas the opposite is true at G2. Since fusion of myoblasts into myotubes occurs during G1 phase, an attractive hypothesis is that the MyoD/Myf5 ratio is an important determinant in myoblasts for the decision process between proliferation or differentiation. In this model, MyoD and Myf5 are more than muscle determination factors, and act in myoblasts as regulators of the proliferation/differentiation interface. Possibly connected to this hypothesis, it is interesting to note that, in cell culture, myoblast differentiation results in two distinct populations: a majority of plurinucleated myotubes that contain high levels of MyoD, but no Myf5, and a minority of quiescent cells, assimilated to "reserve cells", that contain Myf5 but no MyoD [17,18].

Several studies have demonstrated that the cell cycle variations of MyoD and Myf5 levels involve their specific and regulated proteolysis by the proteasome. MyoD degradation at the end of the G1 phase is promoted by its phosphorylation by the CyclinE/CDK2 complex [11,12,19]. Regarding the mechanisms of Myf5 accelerated degradation at the G2/M transition and throughout mitosis, much less is known, although it coincides with the phosphorylation of the protein [20], and seems to depend on the integrity of a Destruction-box (D-box) domain [21].

To test the hypothesis that the cyclic and alternated degradation of MyoD and Myf5 is not a simple consequence of proliferation, but an important event in the control of the proliferation/differentiation interface, we decided to specifically interfere with the mitotic degradation of Myf5 in order to gain insights into the biological role of this process. To this aim, the purpose of the present work was to initiate the characterization of the mechanisms regulating Myf5 mitotic degradation. Since in muscle cells the differentiation process and the cell cycle are tightly connected, we chose to use non-muscle experimental systems in which Myf5 mitotic degradation is conserved and can be studied in a cell cycle-dependent but differentiation-independent manner. We show that in *Xenopus *egg extracts, which are widely used to study mitosis-specific processes, Myf5 mitotic degradation occurs through a ubiquitin- and proteasome-dependent mechanism, in a manner tightly controlled by its phosphorylation, but independent of APC/C activity. Myf5 phosphorylation is itself dependent on the activity of several kinases and phosphatases, and thus depends on a delicate balance between these activities that finely tunes Myf5 stability.

## Results

### Accelerated degradation of Myf5 during mitosis is cell cycle- but not muscle-dependent

In order to better define the parameters that control Myf5 cyclic degradation, we first asked whether the responsible machinery is uniquely dependent on the cell cycle apparatus, or whether it requires components restricted to myogenic cells. To this end, we derived from HeLa cells a stable line expressing Myf5 under an inducible promoter.

**Figure 1:**
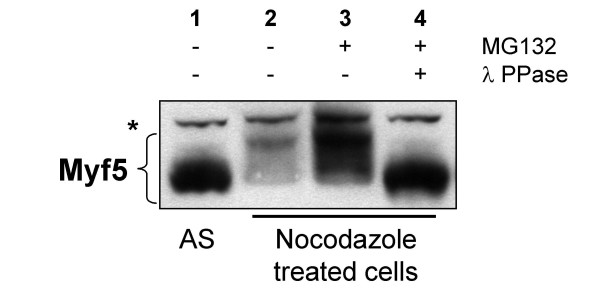
**Myf5 mitotic degradation is conserved in non-muscle cells. **HeLa-S3 cells expressing Myf5 under an inducible promotor (tet^off^) were treated (lanes 2, 3, 4) or not (AS, lane 1) with nocodazole (200 ng/ml) for 16 h, as in [20]. In lanes 3 and 4, cells were treated with a proteasome inhibitor (MG132, 50 μM) for 2 h prior to lysis (as described in Methods). In lane 4, extract was incubated with Lambda phosphatase (New England Biolabs) for 30 min at 30°C. 25 μg of each extract were resolved by 10% SDS-PAGE and immunoblotted with anti-Myf5 antibodies. AS: asynchronous cells; * indicates a non specific band recognized by the antibodies that can be used as a loading control.

In this cell line, induced Myf5 is easily detected in non-synchronized cells, but is absent or very low in cells arrested in mitosis with nocodazole (figure 1, lanes 1 & 2). Treatment with the proteasome inhibitor MG132 significantly increases Myf5 content in non-synchronized cells (data not shown), but has a dramatic effect on Myf5 expression in metaphase cells (figure 1, lane 3), suggesting that Myf5 degradation, which is already rapid in the other phases of the cell-cycle, is strongly accelerated during mitosis. In addition, the forms of Myf5 that are stabilized upon proteasome inhibition in metaphase-arrested cells are phosphorylated, since treatment of the cell extract with lambda phosphatase abolishes the shift seen after electrophoresis without phosphatase treatment (figure 1, lane 4).

These results strictly reproduce the data obtained with similar experiments in myogenic cells, showing that Myf5 is strongly destabilized in mitosis, and is stabilized by proteasome inhibitors as a phosphorylated form [20]. Thus, the machinery responsible for Myf5 mitotic degradation is present in non-myogenic cells, and does not require muscle specific components but only the basal cell cycle machinery present in all dividing cells.

### Myf5 degradation is also cell cycle regulated in Xenopus egg extracts

Since Myf5 mitotic degradation is conserved in non-muscle cells, we decided to test whether we could use *Xenopus *egg extracts as an *in vitro *system to dissect the mechanisms involved. Indeed, *Xenopus *egg extracts recapitulate cell cycle events and have been extensively used for biochemical analyses of mitotic processes, particularly mitosis-specific protein degradation [22], since it is easy to prepare large amounts of extracts that reproduce mitotic or interphase conditions [23]. Compared to extracts of synchronized cultured cells, this system presents in addition the important advantage of limiting dilution of intracellular components and thus preserving the relative activities of enzymes such as protein kinases and phosphatases [23].

**Figure 2:**
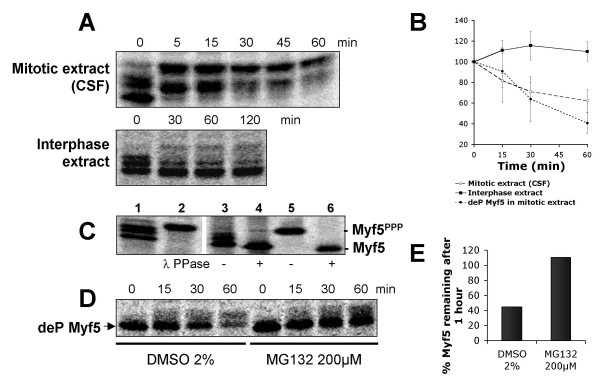
**Myf5 is degraded in a cell-cycle dependent manner in *Xenopus *egg extracts, and its degradation is correlated with changes in its phosphorylation status. **(A) Myf5 is phosphorylated and degraded in mitotic (CSF) extracts, but dephosphorylated and stable in interphase extracts. Degradation assays were performed as described in Methods; 2 μl samples were taken at the indicated times and resolved by 10% SDS-PAGE. (B) Degradation of Myf5 was quantified and normalized (start = 100%) : black squares represent the average degradation of Myf5 in interphase extracts (5 independent experiments), open circles the average degradation of Myf5 in CSF extracts (7 independent experiments), and black circles the average degradation of dephosphorylated (deP) Myf5 (see Methods section) in CSF extracts (4 independent experiments). Bars represent standard deviation. For each lane, a region corresponding to all non-phosphorylated and phosphorylated forms of Myf5 was quantified using the ImageQuant software (Molecular Dynamics). The apparent increase of Myf5 level in interphase extracts is probably due to the accumulation of all the initially phosphorylated forms of Myf5 (some of them being close to background) into the fastest migrating form of Myf5. (C) All the slower migrating forms of Myf5 are phosphorylated: lane 1: *in vitro *translated Myf5; lane 2: *in vitro *translated Myf5 after 1 h incubation with CSF extract in the presence of 1 μM microcystin LR (this treatment leads to hyperphosphorylation of Myf5 (Myf5^PPP^), see text and figure 3 for details); lanes 3–6: *in vitro *translated Myf5 (lanes 3 and 4), or Myf5^PPP ^(lanes 5 and 6) were immunoprecipitated with anti-Myf5 antibodies and incubated in the absence or presence of Lambda phosphatase (λ PPase), as indicated. Samples were resolved by 10% SDS-PAGE and analyzed using a PhosphoImager. (D) A mitotic extract was first incubated with 200 μM MG132, or the same volume of pure DMSO as a control, for 10 min at 25°C; dephosphorylated (deP) Myf5 (see method) was then added to the treated extract. At each time point, 2 μl samples were resolved by 10% SDS-PAGE. (E) Relative amounts of Myf5 in the gel shown in D were quantified as above and reported on the graph.

When radiolabeled *in vitro *translated Myf5 was incubated in mitotic extract (CSF, see Methods section), it was readily degraded, in clear contrast with the stability of the same protein incubated in interphase extract (figures 2A, 2B). As seen in cells, proteasome inhibitors completely stabilize Myf5 (figure 2D, 2E), which does not occur with inhibitors of other proteases (data not shown). Thus the mechanisms governing mitotic-dependent proteasomal degradation of Myf5 are conserved in *Xenopus *egg extracts. It is striking to note that Myf5 appears stable in interphase *Xenopus *extracts, although it is highly unstable in non-synchronized (i.e. mostly interphase) cultured cells [21]. Since in cells, the pathway responsible for Myf5 degradation in interphase is seemingly different from the pathway operating in mitosis [21], one likely explanation is that only the mitotic pathway is active in *Xenopus *extracts. The fact that cell cycles in early embryos lack G1 phase and that *Xenopus *egg extracts do not form nuclei unless sperm heads are added could explain the absence of this interphase pathway, if it requires intact nuclei for example. Thus, *Xenopus *egg extracts appear to be a unique system in which Myf5 mitotic degradation can be analyzed without interference of other (i.e. non-mitotic) pathways, and we decided to pursue our analyses in this system to learn more about the machinery involved specifically in Myf5 mitotic degradation. We note that the mitotic degradation pathway of Myf5 appears distinct from that of cyclin B, which is stable in mitotic extracts (CSF), and degraded only after calcium activation of these extracts [22].

### Myf5 mitotic degradation is controlled by its phosphorylation status

When the fate of Myf5 is analyzed by electrophoresis during incubation in mitotic extracts (figure 2A), there is, concomitant with its gradual disappearance, a clear up-shift in mobility of the protein. By contrast, in interphase extracts, Myf5 is down-shifted and is stable. This strongly suggests that, consistent with the results obtained in mammalian cells, Myf5 mitotic degradation requires its phosphorylation. In view of the heterogeneity of the migration of Myf5 in the gel, we tested whether the different forms correspond to differentially phosphorylated molecules of Myf5. We found that indeed, all the slower migrating forms of Myf5 can be down-shifted to the fastest migrating form upon incubation with lambda phosphatase (figure 2C, lanes 4 and 6). Thus Myf5 degradation is strongly correlated with its extensive phosphorylation.

**Figure 3:**
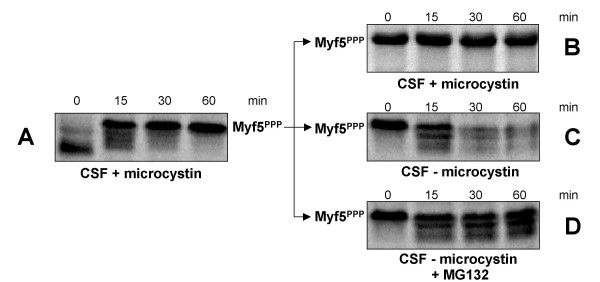
**Hyperphosphorylated Myf5 is stable. **An equal volume of reticulocyte lysate containing *in vitro *translated Myf5 and of *Xenopus *mitotic egg extract (CSF) were incubated together for 60 min in the presence of 1 μM microcystin LR. At each time point, 0.5 μl samples were analyzed by 10% SDS-PAGE (panel A). After this initial incubation, 2 μl of the reaction mixture containing hyperphosphorylated Myf5 (Myf5^PPP^) were further incubated in 18 μl of fresh mitotic extract, either in the presence of 1 μM microcystin (panel B) or without inhibitor addition (panel C), or without microcystin and in the presence of the proteasome inhibitor MG132 (200 μM, panel D). At each time point, 2 μl samples were analyzed by 10% SDS-PAGE.

Interestingly, in extracts degrading Myf5, the protein is gradually shifted to an upper residual form (that we will call thereafter the hyperphosphorylated form of Myf5, see discussion for this terminology) that appears to be stable (figure 2A). By contrast, if Myf5 is dephosphorylated after translation (using alkaline phosphatase bound to agarose beads that can be subsequently easily removed from the reticulocyte lysate), then the protein (deP Myf5) is slower than untreated Myf5 to reach the hyperphosphorylated form when incubated in *Xenopus *egg extracts (compare figures 2A and 2D) and, as a consequence, can be degraded to a greater extent (figure 2B). These observations suggest that Myf5 degradation both requires phosphorylation of the protein, and is inhibited by hyperphosphorylation. To verify this hypothesis, we tested whether the form accumulating upon prolonged incubation of Myf5 in mitotic extracts (i.e. the hyperphosphorylated form of Myf5) is indeed stable. To this end, we first incubated Myf5 in mitotic extracts treated with the phosphatase inhibitor microcystin LR. In these conditions, Myf5 is rapidly accumulated under its hyperphosphorylated form (Myf5^PPP^), and is not degraded (figure 3A). We then incubated this hyperphosphorylated form of Myf5 in a fresh mitotic extract, in the presence or absence of microcystin. As seen in figure 3B, in the presence of microcystin, the hyperphosphorylated form of Myf5 remains unchanged and stable. By contrast, in the absence of microcystin, the hyperphosphorylated form of Myf5 is dephosphorylated and degraded, in a proteasome-dependent manner (figures 3C &3D).

Altogether, our results strongly support the following model: degradation of Myf5 is triggered by its phosphorylation, that occurs in multiple sites, but there is a kinetic competition between phosphorylation and degradation such that, if Myf5 reaches its hyperphosphorylated form, it escapes the degradation machinery. In extracts where both kinases and phosphatases modifying Myf5 are active, such as *Xenopus *mitotic egg extracts, the equilibrium between both types of activity permits some dephosphorylation (and thus subsequent degradation) of Myf5 after it has reached its hyperphosphorylated state, explaining the gradual disappearance of the hyperphosphorylated form seen in figure 2A. By contrast, if phosphatases are inhibited, then Myf5 remains hyperphosphorylated and is strongly stabilized (figures 3A, B).

The differences observed in the phosphorylation pattern of Myf5 between figures 2A and 2D show that, in mitotic *Xenopus *egg extracts, hyperphosphorylation of Myf5 is accelerated by the intervention of active kinases present in the reticulocyte lysate in which Myf5 is translated. However, kinase(s) triggering hyperphosphorylation of Myf5 also exist(s) in mitotic *Xenopus *egg extracts, since dephosphorylated Myf5 rapidly shifts to its hyperphosphorylated form if the extract is treated with the phosphatase inhibitor microcystin (data not shown). This indicates that the stability of Myf5 is tightly dependent upon the kinase/phosphatase equilibrium of the extracts, and suggests that in cells, multiple mechanisms – including perhaps hyperphosphorylation – may be used to control Myf5 stability under different biological conditions.

### Several signaling pathways are likely to be involved in the control of Myf5 stability

**Figure 4:**
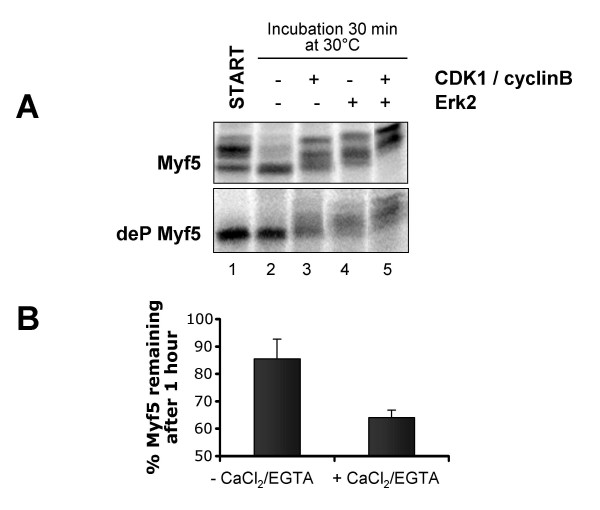
**Several signaling pathways are likely to control Myf5 mitotic degradation **(A) 1 μl of untreated (upper panel) or dephosphorylated (deP, lower panel) Myf5 was incubated with buffer (lane 2), recombinant CDK1/cyclin B (New England Biolabs, 40U, lane 3), recombinant Erk2 (New England Biolabs, 200U, lane 4) or both kinases (lane 5) for 30 min at 30°C. The reaction mix was resolved by 10% SDS-PAGE. "Start" is a non-incubated control. (B) Myf5 degradation is sensitive to very low concentrations of calcium: a mitotic extract was prepared using XB without CaCl_2 _but supplemented with 6 mM EGTA (see Methods), then incubated with 0.1 mM CaCl_2 _and 6 mM EGTA, or an equivalent volume of water as a control, for 10 min at 25°C. 2 μl of Myf5 were incubated for 1 hour with 18 μl of treated extract. 2 μl samples were resolved by 10% SDS-PAGE at the beginning and the end of the reaction. Myf5 degradation rate was quantified as previously described; the graph represents mean values of 3 experiments. Bars represent standard deviations.

An important question is the nature of the kinase(s) responsible for Myf5 mitotic degradation. An evident candidate is the CDK1 kinase, which is the hallmark of mitotic status, since its activity is high during mitosis but abruptly decreases upon mitosis exit, due to the degradation of its activator cyclin B [24,25]. Indeed, *in vitro *translated Myf5 can be directly phosphorylated by CDK1/cyclin B, as seen by the shift of the protein after its incubation with the purified kinase (figure 4A, lanes 1&3). However, if the kinase CDK1 is reactivated in interphase egg extracts by addition of non-degradable cyclin B [26], although phosphorylation of Myf5 resumes in a pattern very similar to that seen in CSF extracts, in most experiments Myf5 is not significantly degraded (data not shown). This suggests that at least one other kinase is required for Myf5 degradation, in addition to CDK1. We observed that Myf5 can nevertheless be degraded in some mitotic extracts resulting from reactivation of CDK1 in interphase extracts (data not shown), pointing to the possible involvement of the MAP kinase family. Indeed, the activity of MAP kinases can vary in interphase *Xenopus *egg extracts, depending on the protocols used to prepare them, which may or may not entail the degradation and/or inactivation of the upstream MAPKKK c-Mos [27]. We found that the protein kinase Erk (MAPK) is also able to phosphorylate Myf5 *in vitro*, apparently at different sites from CDK1/cyclin B (figure 4A, lanes 4&5). These results suggest that these two kinases may cooperate in the regulation of Myf5 stability. However, other enzymes may also be involved. For example, we found that Ca^2+ ^– at very low concentrations – is important for Myf5 degradation, suggesting that a calcium-dependent kinase or phosphatase could be involved in the regulation of Myf5 stability. Indeed, as shown in figure 4B, degradation of Myf5 is accelerated in mitotic extracts (that are prepared in the presence of EGTA) supplemented by a low concentration of Ca^2+^, as compared to degradation in the same extract without supplementation. This reproducible result shows that the concentration of free Ca^2+ ^that exists in the presence of the strong calcium chelator EGTA, albeit extremely low (in the range of 10^-7^M [28]), is nevertheless significant enough to alter the rate of degradation of Myf5. Interestingly, the activity of the calcineurin phosphatase has been shown to be sensitive to such low concentrations of Ca^2+ ^[29]. This range of calcium concentrations does not trigger cyclin B degradation, which requires a transient increase of the concentration of free calcium from 10^-7 ^to 10^-5 ^M for its activation [28].

To define which kinases are required for Myf5 mitotic degradation, we tested a panel of specific inhibitors (see Table 1) targeting the MAP kinase family, the GSK3 kinase, PKC, CDK1/cyclin B, and the calcineurin phosphatase. Unfortunately, we were unable to reproducibly alter Myf5 degradation using these inhibitors, although most of them altered Myf5 phosphorylation status (see Table 1). Overall, the many attempts that we have made to clarify the question of the kinases involved in Myf5 degradation in *Xenopus *extracts have led us to the conclusion that Myf5 degradation probably involves several kinases, but is so sensitive to the kinase/phosphatase equilibrium that alteration of the activity of many signaling pathways induces complex and contradictory effects on Myf5 stability that cannot be clarified by simple pharmacological approaches.

**Table 1:** List of kinase and phosphatase inhibitors tested in *Xenopus *egg extracts for their potential effect on Myf5 degradation These inhibitors were purchased from Sigma-Aldrich, stock solutions were prepared in ethanol or DMSO, following the furnisher instructions.

**Inhibitor**	**Action**	**Concentrations tested**	Effect on Myf5 phosphorylation status (*)	**References**
Indirubin-3'-oxime	GSK3β and CDK5 inhibitor	10^1 ^to 100 μM	Partial inhibition	[54, 55]
Roscovitine	CDK/cyclin inhibitor	100 μM^2^	Partial inhibition	[56–58]
Staurosporine	Broad spectrum kinase inhibitor (among which: PKA, PKG, CaMK, MLCK, PKC)	1^1 ^to 10 μM	Strong inhibition	[59, 60]
U0126	MEK1/2 inhibitor	50^1 ^to 100 μM	Partial inhibition	[61, 62]
GF109203X	PKC inhibitor	5^1 ^to 50 μM	Partial inhibition	[63, 64]
Cyclosporin A	Calcineurin inhibitor	1^1 ^to 10 μg/ml	No effect	[65, 66]
Microcystin LR	Broad spectrum phosphatase inhibitor	1 μM	Hyperphosphorylation	[42, 67]

^1 ^Usual concentration for treatment of cell cultures

^2 ^Efficiency of this concentration was tested by measuring H1 Kinase activity after treatment.

(*) as judged by electrophoretic migration pattern

In addition, it seems likely that although multiple phosphorylations of Myf5 are required for its degradation, none of the individual phosphorylation sites is absolutely necessary. We investigated this question by mutational analyses of Myf5 in order to identify phosphorylation sites important for its degradation. Since Myf5 possesses about 20% serine/threonine (S/T) residues and many potential phosphorylation sites in its primary sequence, making it difficult to systematically change them all into non-phosphorylable residues, we decided to concentrate our work on S/T-proline (S/TP) motifs, which are potential phosphorylation sites for both CDK1/cyclin B and MAPK family kinases. We mutated into alanines the five serines (S_10_, S_23_, S_158_, S_172_, S_231_), and the threonine (T_242_) that are present in Myf5 S/TP motifs, and analyzed the degradation of these mutants in mitotic egg extracts. Although most mutants showed an altered pattern of phosphorylation when compared to the wild type protein, indicating that the mutated residues are likely to be phosphorylated in extracts, none of them was clearly stabilized (not shown). The fact that the mutant S158A, which was found to be stabilized in interphase but not mitotic mammalian cells [21], is still efficiently degraded in mitotic egg extracts, underlines that only the mitotic degradation of Myf5 is active in these extracts. If it cannot be excluded at present that other phosphorylation sites are the key determinants of Myf5 stability, there is another possible interpretation, that we presently favor in view of the probable involvement of both (at least) CDK1/cyclin B and MAP kinases into Myf5 degradation. That is, in a process analogous to that reported for the CDK inhibitor Sic1 in budding yeast [30,31], it would be the number of phosphorylated residues rather than their identity that controls Myf5 stability. If this hypothesis holds true, then only the mutation of most of these sites would significantly alter the degradation of the protein.

### Myf5 degradation requires its prior ubiquitylation

**Figure 5:**
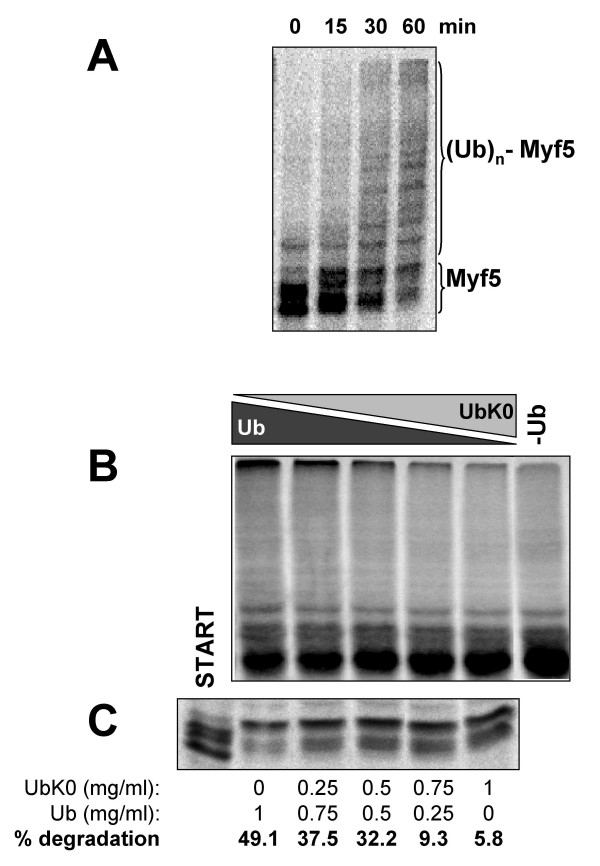
**Polyubiquitylation of Myf5 is required for its degradation. **(A) Myf5 is polyubiquitylated in mitotic extracts: Myf5 was incubated in a mitotic extract in the presence of ubiquitin (1 mg/ml), MG132 (200 μM) and ubiquitin aldehyde (5 μM). At the times indicated, 2 μl samples were analyzed by 10% SDS-PAGE to visualize the formation of high-MW adducts ((Ub)_n_-Myf5, poly-ubiquitylated Myf5 molecules). (B) Lysine-less (K0) ubiquitin inhibits Myf5 polyubiquitylation: Myf5 ubiquitylation was performed in a mitotic extract in the presence of MG132 (200 μM), ubiquitin aldehyde (1 μM), ubiquitin (Ub) and lysine-less-ubiquitin (UbK0) at the indicated concentrations, for 30 min at 25°C. (C) Lysine-less ubiquitin stabilizes Myf5: Degradation assays were performed in 20 μl reaction mixtures containing 16 μl of mitotic extract, 2 μl of radiolabeled *in vitro *translated Myf5 and a mixture of wild type and K0 ubiquitin as indicated. The reaction was performed at 25°C for 40 min, 2 μl samples were resolved by 10% SDS-PAGE. "START" corresponds to a 2 μl sample taken at time 0 from the reaction containing 1 mg/ml Ub. Radioactivity was quantified as previously described (see legend of figure 2) and percentages of degradation were calculated for each Ub/UbK0 ratio from the difference between the times 0 (not shown) and 40 minutes.

Regulated protein degradation by the proteasome most often requires the prior conjugation on the substrate of a poly-ubiquitin chain that acts as a signal targeting the substrate to the proteasome [32]. Incubation of Myf5 in *Xenopus *mitotic egg extracts in the presence of proteasome and de-ubiquitylating enzymes inhibitors indeed led to the rapid formation of ubiquitylated forms of Myf5 (figure 5A). To test whether these ubiquitylated forms are obligatory intermediate products in Myf5 degradation, we titrated wild type ubiquitin (Ub) with a mutant form of Ub in which all the lysines have been replaced by arginines (UbK0). Since this lysine-less mutant cannot be itself conjugated by Ub, it acts as a chain terminator if integrated in a Ub-chain [33]. When added to a mitotic extract, this mutant strongly inhibits both Myf5 ubiquitylation (figure 5B) and degradation (figure 5C) in a concentration-dependent manner. Thus efficient poly-ubiquitylation of Myf5 is required for its subsequent degradation, showing that Myf5 is degraded in a ubiquitin- and proteasome- dependent process.

### The E3 responsible for Myf5 ubiquitylation is not the APC/C

Protein ubiquitylation requires an enzymatic cascade involving 3 types of proteins called E1 (Ub-activating protein), E2 (Ub-carrier protein) and E3 [34]. In this cascade, the E3 component recruits both an E2 and the substrate to favor ubiquitylation of the latter, and thus acts as the specificity factor for the reaction. As a consequence, eukaryotic cells contain a very high number of E3 proteins [35].

However, many substrates degraded by the ubiquitin-proteasome pathway in mitosis (among which the mitotic cyclins and securin) are targeted for ubiquitylation by the E3 ligase APC/C (Anaphase-Promoting Complex / Cyclosome) [36,37]. Ubiquitylation of some of these proteins depends on a loosely conserved Destruction-box (D-box) motif, which is thought to be recognized by the APC/C adaptor proteins Cdc20/Fizzy [25,38] and Cdh1 [39]. A D-box-like motif is present in Myf5 sequence, and substitution of residues into this 9-amino-acid sequence significantly stabilizes the protein during mitosis [21], suggesting that Myf5 could be an APC/C substrate. However, the degradation pathway of Myf5 in mitotic cells appears different with respect of both timing and mechanism from that of known substrates of the APC/C [21]. To elucidate this apparent dilemma, we decided to test in our system whether the APC/C is responsible for Myf5 ubiquitylation.

**Figure 6:**
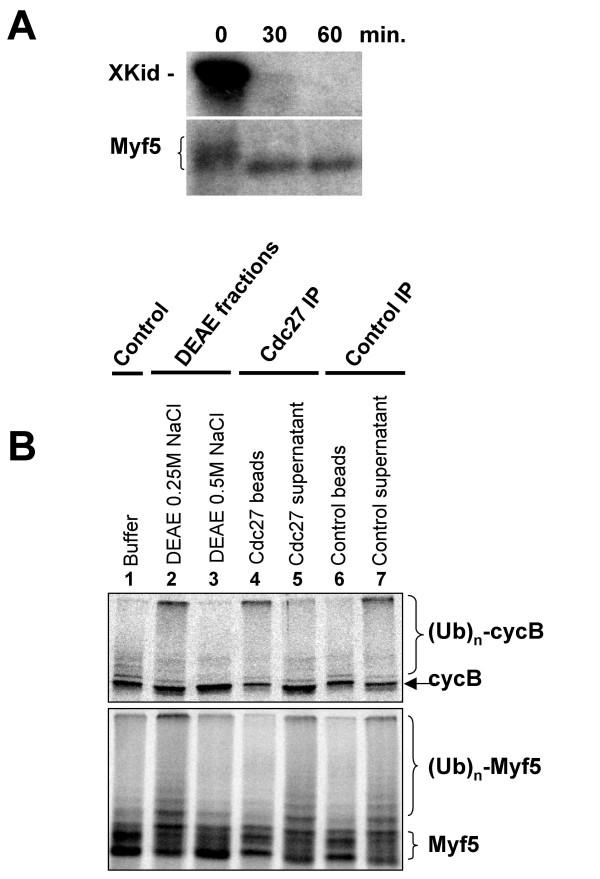
**The E3 responsible for Myf5 ubiquitylation is not the APC/C. **(A) *In vitro *translated Xkid (upper panel) or Myf5 (lower panel) were incubated in 15 μl *Xenopus *interphase egg extract in which Cdh1 has been translated [39]. After the times indicated, 2.5 μl were analyzed by SDS-PAGE and fluorography. (B) 1 ml of mitotic extract was activated with 1 μM microcystin LR (in order to fully activate APC/C), then fractionated on a DEAE column (see Methods). Bound proteins were eluted in two steps with buffer A containing 0.25 M and 0.5 M NaCl, respectively. Each fraction (lanes 2 & 3) was compared to a control reaction containing buffer (lane 1) for its ability to mediate ubiquitylation of either cyclin B (upper panel) or Myf5 (lower panel). Lanes 4–7: the 0.25 M NaCl eluate that mediates both cyclin B and Myf5 ubiquitylation was then subjected to immunoprecipitations using either anti-Cdc27 or control antibodies. For each immunoprecipitation, both the material bound to the beads or the supernatant were analyzed for ubiquitylation activity using cyclin B (upper panel) or Myf5 (lower panel) as a substrate. (Ub)_n_-cycB and (Ub)_n_-Myf5 indicate poly-ubiquitylated forms of cyclin B and Myf5, respectively.

We first used *Xenopus *interphase extracts in which APC/C is specifically activated by translation in the extract of the APC/C regulator Cdh1 (Fizzy-related in *Xenopus*), as these extracts have been shown to be able to efficiently degrade most known APC/C substrates in a Cdh1-dependent manner [39]. As shown in figure 6A, Myf5 is rapidly dephosphorylated and is stable for 1 h in these extracts, although the APC/C substrate Xkid [40], used here as a control, is completely degraded after 30 minutes, as expected. This result strongly suggests that APC/C is not involved in Myf5 degradation. However, Myf5 could be an unusual APC/C substrate requiring to be phosphorylated to be recognized by this E3. If so, then its dephosphorylation in these extracts could explain its stability. To avoid dephosphorylation, we decided to test by immunodepletion approaches whether the presence of APC/C is required for Myf5 ubiquitylation in mitotic eggs extracts. In these extracts, APC/C is active against certain substrates such as cyclin A [41], but its activity towards other substrates such as cyclin B is inhibited by the cytostatic factor (CSF). In order to abolish CSF effect and study a fully active APC/C, we treated a CSF extract with 1 μM microcystin LR [42]. Since in these conditions, as described above, Myf5 degradation is inhibited due to the accumulation of the protein in its hyperphosphorylated form, we fractionated the activated extract on a DEAE column. Both APC/C and E3 acting on Myf5 were eluted with 0.25 M NaCl (figure 6B, lanes 2). Immunodepletion of APC/C, using antibodies directed against its constitutive subunit CDC27, fails to abolish Myf5 ubiquitylation in the 0.25 M NaCl fraction, whereas, in the same conditions, cyclin B is no longer ubiquitylated (figure 6B, lanes 5). Moreover, cyclin B, but not Myf5, is ubiquitylated in the presence of the proteins immunoprecipitated with anti-CDC27 antibodies (figure 6B, lanes 4). Altogether, these results show that, at least in *Xenopus *egg extracts, the E3 responsible for Myf5 ubiquitylation in mitosis is not the APC/C, but another E3 that remains to be identified.

### The E3 involved in Myf5 ubiquitylation preferentially recognizes phosphorylated forms of Myf5

**Figure 7:**
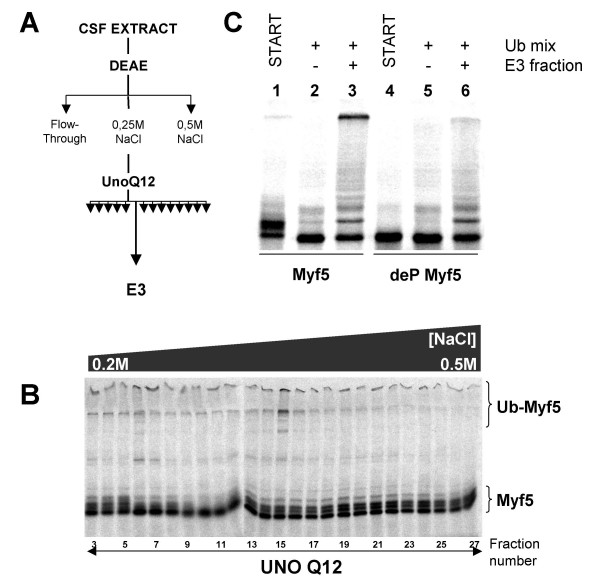
**Myf5 ubiquitylation is controlled by phosphorylation. **(A) Scheme of Myf5 E3 purification : for details see Methods section. (B) 2 ml of mitotic extract were fractionated as described in A; the fractions obtained from the UnoQ column were screened for their ability to ubiquitylate radiolabeled Myf5 in the presence of a ubiquitylation mix containing GST-Ub (see Methods). The reactions were resolved by 10% SDS-PAGE. (Ub)_n_-Myf5 indicates poly-ubiquitylated forms of Myf5. (C) The fraction containing the E3 was concentrated about 10-fold using a Centricon 10 K (Millipore). Untreated Myf5 (lanes 1 to 3) or dephosphorylated (deP) Myf5 (lanes 4 to 6) was incubated with the ubiquitylation mix alone (lanes 2, 5) or together with 6 μl of concentrated fraction containing the E3 (lanes 3, 6) for 30 minutes at 25°C. Non incubated Myf5 was loaded in lanes 1 and 4 (START) as control. Similar amounts of radioactive Myf5 and dephosphorylated Myf5 were used.

To further characterize the E3 responsible for Myf5 ubiquitylation, we undertook fractionation of mitotic egg extracts by chromatography (figure 7A). In this type of experiments, the fractions containing the E3 activity of interest are identified by their ability to allow ubiquitylation of Myf5 when added to a reaction mixture containing purified E1, E2, Ub and radiolabeled Myf5. The E3 activity was retained on a DEAE column, as described above (figure 6B, lane 2), and quantitatively recovered within the proteins eluted from the column with 0.25 M NaCl. Further fractionation of the DEAE 0.25 M NaCl eluate on a UnoQ column reproducibly led to the ubiquitylation pattern shown in figure 7B: a weak ubiquitylation activity could be detected in several fractions, but the major activity resulted from proteins eluted in fraction 15 (about 0.3 M NaCl). We failed to observe a robust ubiquitylation at this stage, probably because we removed from the active fractions the kinases important for Myf5 ubiquitylation, but also because the E3 appeared to be very unstable. Indeed its activity was essentially lost a few hours after UnoQ purification (data not shown). However, we used freshly prepared E3 fractions to test whether this E3 discriminates between phosphorylated and non-phosphorylated forms of Myf5, as suggested by our previous analyses in crude extracts. We took advantage of the fact that Myf5 is already phosphorylated during its translation in reticulocyte lysate and tested the fraction from the UnoQ column containing the E3 activity, for its ability to mediate ubiquitylation of either untreated (phosphorylated) Myf5 or Myf5 dephosphorylated (deP Myf5) by treatment of the lysate with alkaline phosphatase. Figure 7C shows that ubiquitylation of Myf5 occurs when using the phosphorylated form of Myf5, but is strongly diminished (although not abolished) when using non-phosphorylated Myf5. This result confirms that phosphorylation of Myf5 favors its recognition by the E3 mediating its ubiquitylation.

## Discussion

The striking differential cell cycle-regulated degradation of both MyoD and Myf5 in proliferating myoblasts suggests that these two proteins, albeit homologous and partially redundant, carry out specific functions that must be turned down at a specific cell cycle stage. Physical and functional interactions of MyoD with cell cycle regulators, that impinge on the proliferation/differentiation interface in myoblasts, show that certain of these functions are interfering with cell cycle progression and suggest that, at least for MyoD, its cyclic degradation is required for maintenance of the proliferative status of the cells. For Myf5, much less is known, but it is likely that the same rationale can be followed, i.e. that its accelerated degradation at the late G2/M phase of the cell cycle is required for progression through subsequent phases.

### Nature of the E3 responsible for Myf5 mitotic ubiquitylation

A strong argument in favor of an involvement of APC/C in Myf5 mitotic degradation was that Myf5 contains in its sequence a putative D-box motif, the mutation of which partially stabilizes Myf5 in mitotic cells, but not in interphase cells [21]. However, this degradation did not depend on UbcH10, the E2 known to function with APC/C, and the timing of degradation of Myf5 appeared different from that of known substrates of the APC/C: when examined in individual cells, Myf5 was always degraded before cyclin B, but the timing of its degradation relative to that of cyclin A appeared highly variable [21]. In addition, it is noteworthy that ubiquitylation of known substrates of the APC/C is not regulated by phosphorylation of the substrate [43]; the fine temporal tuning of ubiquitylation is rather controlled by post-translational modifications of the APC/C itself, respectively the core complex [44] or its activators [45], which modulate the activity of the E3. Altogether, the involvement of APC/C in Myf5 mitotic degradation remained an open issue.

Since mitotic degradation events are usually conserved between cells and organisms, the *Xenopus *egg extract system has been widely used to dissect these events and particularly to study the functions of APC/C [36,46,47]. To resolve the dilemma relative to the possible involvement of APC/C in the mitotic degradation of Myf5, we tested whether this complex is important for Myf5 mitotic degradation in *Xenopus *extracts. We found by two different approaches that APC/C does not participate to the ubiquitylation of Myf5 in *Xenopus *egg extracts. This result raises two questions. The first is whether the *Xenopus *system is representative of other cells and particularly myoblasts as far as Myf5 mitotic degradation is concerned. At this point, we believe that there is no reason to doubt it, as it would be a surprise if Myf5 was degraded in this system in a mitotic-dependent but different manner to that in cultured cells. Since the mechanisms controlling mitosis are conserved in higher eukaryotes, the frog system has been central for the understanding of many mechanisms controlling mitosis (including the discovery of APC/C) and, up until now, most data obtained with *Xenopus *egg extracts for APC/C substrates have been confirmed in other systems. But obviously a definitive conclusion on the role of APC/C in Myf5 degradation will require further studies in myoblasts. The second question is why, if APC/C is not important for Myf5 mitotic degradation, the Destruction-box mutants of Myf5 were specifically stabilized in cells during mitosis [21]. We are presently not able to answer this question, but several observations on this issue may be important. First, the D-box is a loosely conserved motif that can be found in many proteins, and several examples show that it cannot be automatically assimilated to a signature for targeting to the APC/C (see for example [40]). Thus, Myf5 D-box could be important for Myf5 mitotic degradation without actually acting as a genuine D-box motif. Second, there are several degradation pathways acting on Myf5 in cells, and one cannot exclude that the Destruction-box mutants of Myf5 interfere with other systems than the strictly mitotic pathway. In support of this notion, it is important to note that these mutants are only partially stabilized in mitotic cells: their further stabilization by proteasome inhibitors indeed indicates that proteasomes are still actively degrading these mutants in mitotic cells [21]. Moreover, we found no stabilization of these mutants in *Xenopus *egg extracts (data not shown), which, as shown by the absence of degradation of Myf5 in interphase, seem to possess only the mitotic pathway acting on Myf5. Third, because the D-box motif of Myf5 is adjacent to the DNA binding domain, these mutants have a decreased affinity for DNA as compared to the wild type protein [48]. Since binding to DNA and presence of various partners have been shown to alter MyoD degradation [49,50], it is possible that conformational changes induced by mutations in the D-box motif of Myf5 impact on its mitotic degradation by indirect ways.

Altogether, we believe that our data in *Xenopus *are a solid, albeit non definitive, argument in favor of the non-involvement of APC/C in Myf5 mitotic degradation. Interestingly, there are up to now few substrates known to be degraded in mitosis by the ubiquitin proteasome pathway, and whose ubiquitylation is not due to the APC/C [51]. The high incidence of the phosphorylation status of Myf5 on its ubiquitylation and degradation suggests that an E3 from the SCF family of complexes could be involved [43,52]. Since few tools are available to study SCF complexes in *Xenopus *egg extracts, we are currently analyzing the potential involvement of these complexes in Myf5 ubiquitylation using mammalian systems.

### Control of Myf5 degradation by phosphorylation

Based on the homology of Myf5 to MyoD, we expected a simple mechanism in which a unique phosphorylation would trigger Myf5 mitotic degradation. Indeed phosphorylation of MyoD on its serine 200 by the CDK2/cyclin E kinase has been shown to be critical to entail rapid degradation of this protein at the end of the G1 phase of the cell-cycle [12]. However, although this simple scenario cannot be excluded at the moment, our results on the mechanisms controlling Myf5 stability are drawing a much more complex picture.

We found a significant degree of variability from extract to extract in the phosphorylation status of Myf5, that prevented us from obtaining clear conclusions on the nature of the kinase(s) controlling Myf5 ubiquitylation and degradation. We believe that some of the problems were due to the origin of the substrate we used. Indeed, there is a clear interference in this assay of kinases present in the reticulocyte lysate in which Myf5 was translated, and we observed a variability between different lysates that was likely to contribute to the difficulty of obtaining solid conclusions. However, attempts to translate Myf5 in other systems derived from wheat-germ or *E. coli*, or to co-translate the Myf5 partner E12 did not fundamentally solve the problem of variability.

We thus think that the problem resides elsewhere, in the complexity of Myf5 phosphorylation that makes the degradation of this protein extremely sensitive to the kinase/phosphatase equilibrium of the extracts. The concordant picture arising from all our attempts to modify Myf5 stability in *Xenopus *egg extracts, either by inhibition of kinases or phosphatases or by single mutations of the S/TP sites of Myf5, is that several enzymes impinge on Myf5 phosphorylation status and thus Myf5 stability, and that Myf5 ubiquitylation apparently requires phosphorylation on multiple sites. However, although most S/TP sites of Myf5 seem phosphorylated in mitotic extracts, as their mutation affects Myf5 migration in gel, none of these sites seems to be absolutely necessary for efficient degradation of the protein. This could suggest that it is phosphorylation itself or, by analogy with data obtained for the CDK inhibitor Sic1 [30,31], the number of phosphorylated residues rather than their identity that is the important parameter for Myf5 ubiquitylation.

An unexpected result in our experiments was the observation that a phosphorylated form of Myf5 was completely resistant to degradation. Because this form was the latest phospshorylated form to appear and the slowest migrating in gel, we called it the hyperphosphorylated form of Myf5. This terminology is coherent with the observation that Myf5 apparently escapes degradation only when it is fully phosphorylated. However at this point we cannot exclude that a single phosphorylation event is responsible for both Myf5 stabilization and shift to the slowest migrating form. An important question is whether the stabilizing hyperphosphorylation of Myf5 exists in cells and particularly in myoblasts. A possibility is that this hyperphosphorylation is not a standard process seen at each cell cycle, but a regulated mechanism occurring only in certain physiological situations. Alternatively, as it has been suggested recently for MyoD [13], such a mechanism could allow a low amount of Myf5 to be preserved during passage through mitosis, in order for the cell to be able to mobilize it immediately after completion of cell division.

## Conclusion

**Figure 8:**
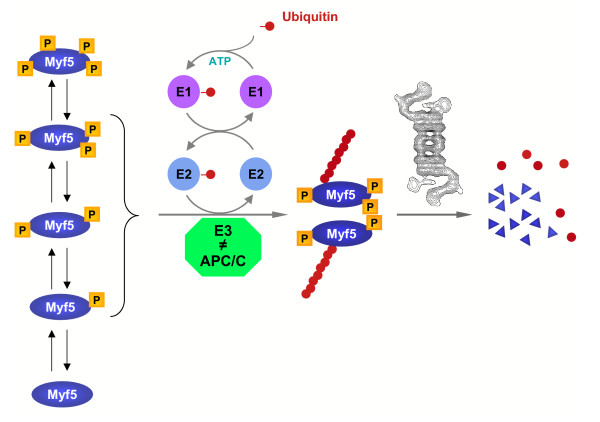
**A model for Myf5 mitotic degradation pathway. **Myf5 is subjected to multiple phosphorylations in *Xenopus *mitotic egg extracts, that tightly control its stability: phosphorylation of the protein leads to its ubiquitylation by an E3 distinct from APC/C, and its subsequent degradation by the proteasome. However, a hyperphosphorylated form of Myf5 remains stable (see text for details).

In this article, we describe the work we performed using the *Xenopus *egg extract system to better define the mechanisms that control Myf5 degradation in mitosis. Altogether, our data are in favor of the following model (figure 8): in mitotic extracts, Myf5 is phosphorylated on numerous residues. Some of these residues, once phosphorylated, are recognized by an E3, distinct from APC/C, that mediates Myf5 polyubiquitylation, targeting it to the proteasome for degradation. However, when Myf5 is fully phosphorylated, it somehow escapes degradation and is stabilized. In such a model, phosphorylation of Myf5 is necessary for its mitotic ubiquitylation, but can also be used to stabilize the protein. Thus the stability of the protein can be finely tuned by differential activation of various signaling pathways, providing a precise and rapid way to adapt Myf5 levels in cells in function of the cell cycle stage.

## Methods

### Cell culture and lysates

HeLa-S3 cells expressing mouse Myf5 (UN6 clone) under an inducible promoter (tet-off) are usually cultured in DMEM containing 10% FCS (Cambrex), 1000 U/ml penicillin/streptomycin, 2 mM L-Glutamine, 500 μg/ml G418, 170 U/ml hygromycin B and 1 μg/ml tetracycline. Expression of Myf5 is induced for 24 hours by washing the cells three times with PBS and adding fresh medium without tetracycline. To prepare lysates, cells are scraped in cold PBS and centrifuged for 10 min at 1000 rpm. The pellet is then resuspended in about 5 pellet volumes of 20 mM Tris pH 7.5, 137 mM NaCl, 10% glycerol, 1% NP40, 1 mM orthovanadate, 20 mM NaF, protease inhibitor mix (Complete, Roche-Boehringer), and incubated for 30 min on ice, while vortexing every 5 min. The lysate is then centrifuged for 8 min at 16000 × g. The supernatant is removed and protein concentration is measured using BSA as a standard (Bradford Reagent Assay, Pierce).

### Xenopus egg extracts

Mitotic extracts are prepared by gently crushing oocytes arrested in metaphase of meiosis II, with CDK1/cyclin B kinase activity maintained at a high level by cytostatic factor (CSF); these extracts, called CSF extracts, thus reproduce a pseudo-mitotic state. When eggs are fertilized, penetration of sperms induces a calcium wave that inactivates CSF activity and triggers cyclin B degradation, and CDK1 activity rapidly declines to an interphase level (reviewed in [53]). A synchronous release from CSF arrest can be artificially induced by activating the eggs with calcium ionophore treatment. To prepare extracts, eggs are dejellied in a solution containing 2% L-Cystein (pH 7.5), then washed extensively in XB Buffer (100 mM KCl, 0.1 mM CaCl_2_, 1 mM MgCl_2_, 10 mM HEPES pH 7.7, 50 mM Sucrose) supplemented with 6 mM EGTA and lysed by centrifugation for 20 min at 16000 × g. The supernatant (except the floating lipidic phase) is removed and supplemented with an ATP regenerating system (1.25 mM ATP, 1.25 mM MgCl_2_, 1.9 mM Creatine Phosphate, 6.25 μg/ml Creatine Phosphokinase), leupeptin (25 μg/ml) and cytochalasin B (25 μg/ml). After centrifugation for 20 min at 16000 × g, the supernatant is removed, aliquoted and frozen in liquid nitrogen. Frozen aliquots are kept at -80°C for several weeks. For interphase extracts, eggs were dejellied as described above, then activated by calcium ionophore and incubated for 10 minutes in XB containing 10 μg/ml cycloheximide (SIGMA-ALDRICH). Eggs were then lysed as described above.

### *In vitro *production of [^35^S] methionine-labeled proteins

Human cyclin B, mouse Myf5 and frog Xkid purified plasmids are transcribed and translated *in vitro *in the presence of L-[^35^S]-methionine (Amersham, Redivue mix) in rabbit reticulocyte lysates, following manufacturer's instructions (Promega TNT Quick coupled system), then loaded on pre-equilibrated (Tris-HCl 20 mM, pH7.5) Biospin6 columns (BioRad), in order to eliminate non-incorporated radioactive methionine. The dephosphorylated form of Myf5 is obtained by incubating freshly translated Myf5 with alkaline phosphatase linked to agarose beads (SIGMA-ALDRICH) for 30 min at 37°C. The mix is then loaded on BioSpin6 columns as above to remove both the beads and the excess radioactive methionine.

### *In vitro *degradation and ubiquitylation assays

For degradation assays, 18 μl of mitotic or interphase extracts are usually mixed with 2 μl of reticulocyte lysate containing the radioactive (*in vitro *translated) substrate and are further incubated at 25°C. At appropriate time points, 2 μl samples are subjected to SDS-PAGE (10% gels) analysis. Gels are then dried, exposed and the radioactive bands are quantified using a PhosphoImager (Molecular Dynamics). Ubiquitylation assays are performed in the presence of 200 μM MG132 (BioMol), 5 μM Ubiquitin aldehyde (BioMol), 1 mg/ml Ubiquitin (SIGMA-ALDRICH), 5 mM MgCl_2 _and 1 mM ATPγS. Lysineless mutant ubiquitin (UbK0) is purchased to Boston Biochem.

### Purification of Myf5 E3

A mitotic extract is diluted twice in buffer A (20 mM Tris pH7.5, 1 mM DTT) supplemented with 1% Igepal CA-630 (Sigma), then incubated for 15 min at 4°C with the same volume of AffigelBlue DEAE beads (BioRad) pre-equilibrated in buffer A + 0.5% Igepal. The beads are then extensively washed with a large excess of buffer A containing 0.5% Igepal, then with buffer A only. Myf5 E3 activity is eluted with buffer A containing 0.25 M NaCl. This fraction is directly loaded on a UnoQ1 or a UnoQ12 column (BioRad) pre-equilibrated with buffer A containing 0.2 M NaCl. A gradient from 0.2 M to 0.5 M of NaCl is applied to the column, and Myf5 E3 activity is eluted at a concentration of approximately 0.3 M NaCl. The fractions obtained from the different columns are screened for their ability to ubiquitylate radiolabeled Myf5 in the presence of the ubiquitylation enzymes E1 (Xenopus, 50 ng), E2 (recombinant UBCH5B, 0.5 μg), purified recombinant GST-Ub (1 mg/ml) or ubiquitin (1 mg/ml), 1 mM ATP, 5 mM MgCl_2_, 5 μM ubiquitin aldehyde, 200 μM MG132, 20 mM Tris pH7.5. Ubiquitylation reactions are performed with 1 μl of radiolabeled Myf5, 6 μl of each fraction, in a final volume of 10 μl, and incubated for 30 min at 25°C. After appropriate incubation time, the reaction is stopped by addition of sample buffer, and the samples are analyzed by electrophoresis and PhosphoImaging.

### Antibodies

Anti-Myf5 (C-20, Santa Cruz) is diluted 1:1000 for Western Blotting. Secondary anti-rabbit-HRP (Amersham) is diluted 1:10000. APC/C immuno-precipitations are realized with a home-made affinity-purified anti-Cdc27 [46].

## Authors' contributions

CD was in charge of this project, and conducted most of the experiments shown in this article. GJG has critically contributed to this work by establishing its experimental basis in Xenopus egg extracts, and by demonstrating stabilization of Myf5 by hyperphosphorylation. CL participated in the conception of the study and established the HeLa cell line expressing Myf5. TL has been a continuous interlocutor during the whole work, and provided his expertise and important tools to analyze Myf5 and Cyclin B degradation in Xenopus egg extracts. GL prepared and tested CSF extracts, and produced various ubiquitylation enzymes. CP participated in the conception of the study. OC conceived the study, participated in its design and coordination. CD and OC wrote the manuscript, with special help of GJG and CL. All authors read and approved the final manuscript.
